# Examination of Organic Vapor Adsorption onto Alkali Metal and Halide Atomic Ions by using Ion Mobility Mass Spectrometry

**DOI:** 10.1002/cphc.201700747

**Published:** 2017-09-18

**Authors:** Anne Maiβer, Christopher J. Hogan

**Affiliations:** ^1^ Department of Mechanical Engineering University of Minnesota Minneapolis MN USA; ^2^ The Cyprus Institute Aglandjia Cyprus

**Keywords:** gas-phase adsorption, ion-induced nucleation, ion mobility, ion–molecule reactions, mass spectrometry

## Abstract

We utilize ion mobility mass spectrometry with an atmospheric pressure differential mobility analyzer coupled to a time‐of‐flight mass spectrometer (DMA‐MS) to examine the formation of ion‐vapor molecule complexes with seed ions of K^+^, Rb^+^, Cs^+^, Br^−^, and I^−^ exposed to *n*‐butanol and *n*‐nonane vapor under subsaturated conditions. Ion‐vapor molecule complex formation is indicated by a shift in the apparent mobility of each ion. Measurement results are compared to predicted mobility shifts based upon the Kelvin–Thomson equation, which is commonly used in predicting rates of ion‐induced nucleation. We find that *n*‐butanol at saturation ratios as low as 0.03 readily binds to all seed ions, leading to mobility shifts in excess of 35 %. Conversely, the binding of *n*‐nonane is not detectable for any ion for saturation ratios in the 0–0.27 range. An inverse correlation between the ionic radius of the initial seed and the extent of *n*‐butanol uptake is observed, such that at elevated *n*‐butanol concentrations, the smallest ion (K^+^) has the smallest apparent mobility and the largest (I^−^) has the largest apparent mobility. Though the differences in behavior of the two vapor molecules types examined and the observed effect of ionic seed radius are not accounted for by the Kelvin–Thomson equation, its predictions are in good agreement with measured mobility shifts for Rb^+^, Cs^+^, and Br^−^ in the presence of *n*‐butanol (typically within 10 % of measurements).

## 1 Introduction

Ion‐induced nucleation^1^,^2^ occurs when it is energetically favorable for vapor molecules to adsorb repeatedly onto ions, growing them substantially in size (into droplets). The study of ion‐induced nucleation is of fundamental importance in understanding condensed phase species formation from vapor^3^, ^4^, ^5^, ^6^ and also finds application in the design of condensation based detection systems (i.e. for analytes in the vapor phase^7^, ^8^, ^9^). Classical models of ion‐induced nucleation, which incorporate the Kelvin^10^,^11^ and Thomson effects^12^ to evaluate the vapor pressure of a small droplet, can be used to predict both ion induced nucleation rates and activation efficiencies for vapor molecule‐ion complexes; however, such predictions are not in agreement with all experimental measurements.^6^,^13^, ^14^, ^15^, ^16^ Most notably, classical Kelvin–Thomson‐based models can explain neither observed dependencies on the sign of the ion,^3^ nor observed dependencies on the ion chemical composition.^4^,^17^, ^18^, ^19^, ^20^, ^21^

Model predictions in ion‐induced nucleation are heavily dependent upon the properties of the so‐called critical cluster,^22^ that is, the ion‐vapor molecule complex of maximum free energy, which is typically in the nanometer to subnanometer size range and is composed of a limited number of vapor molecules. To better understand why discrepancies arise between classical predictions and measurements, it is also desirable to probe the properties of ion‐vapor molecule complexes at the size scale of critical clusters.^16^,^23^,^24^ However, the majority of experimental approaches to examine ion‐induced nucleation rely upon detection of nucleated droplets significantly larger than the critical size,^22^,^25^, ^26^, ^27^ with nucleation theorem based extrapolation applied to infer properties of critical clusters. Distinct from these techniques is ion mobility mass spectrometry,^28^, ^29^, ^30^ which, via doping drift gases with organic vapor molecules, has recently been employed to examine ion‐vapor molecule complexes.^19^,^20^,^31^, ^32^, ^33^, ^34^ Though vapor molecules typically desorb from seed ions in mass spectrometer inlets, during ion mobility measurement, which takes place at controlled pressure and temperature, ions and the surrounding vapor molecule are in equilibrium with one another. Measurement of shifts in an ion's mobility with changes in vapor saturation ratio can then be used to infer the extent of vapor molecule adsorption.^19^,^31^ As vapor dopant concentrations are below saturation during ion mobility measurements, such experiments are the converse to the traditional manner in which ion‐induced nucleation is examined; traditionally micrometer sized droplets (supercritical sizes) formed under supersaturated vapor conditions are probed, while in ion mobility‐mass spectrometry nanometer scale complexes (subcritical sizes) are studied. At the same time, though vapor concentrations are below saturation, they can be higher than are achievable in high pressure‐mass spectrometry, a technique which has been used previously in examined ion‐vapor molecule complexes formed in subsaturated conditions.^35^, ^36^, ^37^ Therefore, ion mobility‐mass spectrometry measurements are well suited to provide information on the earliest stages of ion‐vapor molecule complex formation.

To date, studies utilizing ion mobility‐mass spectrometry to examine ion‐vapor molecule complexes have been focused on proof‐of‐concept measurements,^32^,^33^ the development methods to analyze and interpret results,^31^ examination of how complex formation influences instrument calibration,^38^ and the examination of water and alcohol uptake by salt cluster ions.^19^,^20^ Though the latter are of interest in understanding ion‐induced nucleation, comparison to theoretical predictions is complicated by the possibility that salt cluster ions may partially or wholly dissociate upon vapor molecule adsorption (as is suggested by computational predictions^19^). This has an influence on the ion‐vapor molecule complex free energy (solvation energy), and is difficult to quantify without the use of computational approaches specific to the cluster ion and vapor molecule under examination. A simpler examination of vapor uptake would involve the use of atomic ions as seeds for vapor adsorption, for which dissolution or changes in ion conformation upon vapor adsorption need not be considered. The purpose of this study is to perform measurements along these lines. Specifically, we utilize an atmospheric pressure differential mobility analyzer coupled to a mass spectrometer (DMA‐MS) to examine the formation of alkali metal cation complexes with *n*‐butanol and *n*‐nonane (which have been utilized prevalently in ion induced nucleation/condensation experiments^39^,^40^), as well as halide anion complexes with the noted organic species. Results are compared to modified classical predictions using the analysis framework described by Oberreit et al.^18^,^19^ and Rawat et al.,^31^ linking the shift in mobility/collision cross section (inferred from mobility measurements) brought about by vapor molecule adsorption to the equilibrium sorption coefficients for successive adsorption events.

## Experimental Section

The DMA‐MS system is described in detail in prior studies.^19^, ^20^, ^41^, ^42^, ^43^ Briefly, it consists of a parallel‐plate DMA (P5, SEADM, Boecillo, Spain, with a resolving power in excess of 50) coupled with a QSTAR XL quadrupole‐time‐of‐flight mass spectrometer (MDS Sciex). Atomic ions were generated via electrospray ionization of 10 mm methanol solutions of potassium, rubidium, and cesium iodide salts, as well as tetraheptylammonium bromide (purchased from Sigma–Aldrich, St. Louis, MO, USA). Positive mode was employed to generate cations, and negative mode was employed for anions. Electrospray ionization of salt solutions generates primarily singly and multiply charged cluster ions.^44^ Here, we focus only on measurement of the atomic cations/anions produced. Ions were drawn into the DMA electrostatically against a 0.2 L min^−1^ counterflow of ultrahigh purity air (Airgas). The DMA sheath flow was also ultrahigh purity air, and was maintained at a temperature in the 303–305 K range via application of a water based heat exchanger. For mobility measurements, the potential difference across the DMA was scanned from 500 to 2500 V in 10 V increments. Mass spectra were recorded at each voltage step using the time‐of‐flight section of the mass spectrometer. Controlled amounts of *n*‐butanol and *n*‐nonane vapor were introduced into the DMA sheath using a constant output nebulizer described previously.^18^, ^19^ Prior to all measurements, the entire system was allowed to operate for more than two hours, to ensure thermal equilibration of the DMA sheath flow and that vapor concentration profiles within the DMA were uniform. Between measurements, the DMA‐MS system was not used for any other experiments, in order to minimize the potential for contamination from other chemicals. The compounds *n*‐butanol and *n*‐nonane were chosen for several reasons. First, they have been examined in prior ion‐induced nucleation experiments^39^, ^40^ with clusters/particles in the nanometer size range. Second, *n*‐butanol is prevalently used in condensation particle counters,^9^, ^17^ which are commercially available devices used to detect ions/nanoparticles in the gas phase via condensation of *n*‐butanol onto analytes (growing them to sizes detectable via light scattering). Third, these solvents, of clearly disparate molecular structure, have similar saturation vapor pressures at 304 K (1.3 kPa for *n*‐butanol and 0.6 kPa for n‐nonane) and similar surface energy densities (0.024 J m^−2^ for *n*‐butanol^45^ and 0.023 J m^−2^ for *n*‐nonane). Shown subsequently, classical theory predictions of the extent of uptake are dependent upon the saturation vapor pressure (defining the saturation ratio) and the surface energy density, hence it is of interest to examine solvents with similar bulk properties yet distinct molecular structures.

To quantify vapor uptake by ions, the potential difference in the DMA required to maximally transmit each examined cation and anion was monitored as a function of saturation ratio. In differential mobility analysis, the potential difference is linearly proportional to the inverse mobility of the ions transmitted.^46^ DMA calibration was performed both in the absence and in the presence of organic vapor by determining the voltage required to transmit the tetraheptylammonium ion, whose inverse mobility (1.03 V s cm^−2^) was measured in air at atmospheric pressure by Ude and Fernandez de la Mora.^47^ As noted in several studies^31^,^38^ and also observed here, this ion's mobility appears insensitive to saturation ratio (the voltage required to maximally transmit it does not vary substantially) and it does not appear to form complexes with either of the vapor molecule types examined in the test saturation ratio range.

## 2 Results and Discussion

### 2.1 Ion‐Vapor Molecule Complex Mobilities

**Figure 1:**
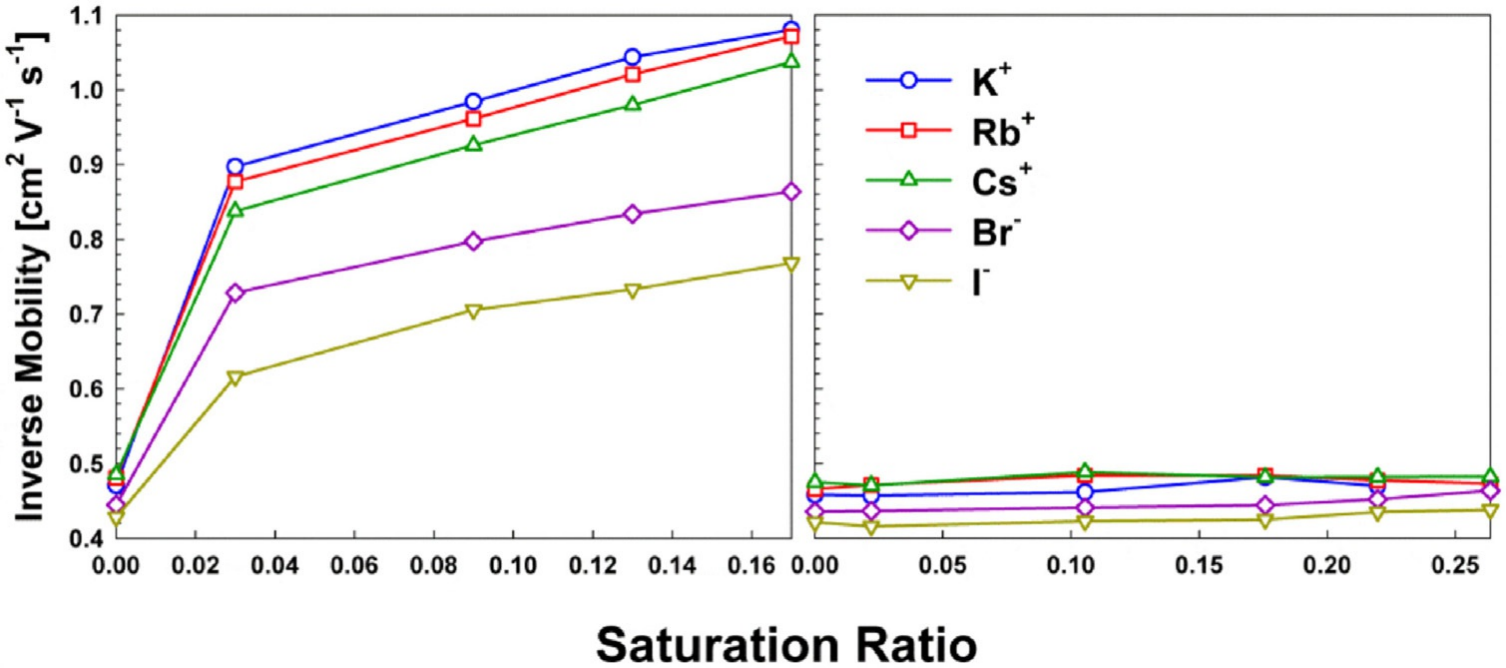
The inverse mobilities of atomic ions as a function of saturation ratio in the presence of a) *n*‐butanol vapor and b) *n*‐nonane vapor, at atmospheric pressure and 304 K.

In total, we made measurements of the inverse mobilities of K^+^, Rb^+^, Cs^+^, Br^−^, and I^−^ at 304 K and atmospheric pressure in air, with butanol saturation ratios in the 0‐0.17 range and nonane saturation ratios in the 0–0.27 range (similar saturation ratio ranges were accessible because of the similar saturation vapor pressures of these two solvents). Inverse mobility is proportional to the apparent collision cross section of the ion under measurement conditions, hence larger inverse mobilities correspond to larger ions (i.e. larger ions have smaller mobilities). The inverse mobilities of the formed ion‐vapor molecule complexes are plotted in Figure 1 a for *n*‐butanol and Figure 1 b for *n*‐nonane, respectively. During transit through the DMA, it is important to note that the number of vapor molecules bound within an ion‐vapor molecule complex is not a constant; each complex is in equilibrium with its surroundings and probes the equilibrium distribution of vapor molecules bound (which is a function of the vapor molecule sorption and desorption rates).^18^ Therefore, the measured inverse mobilities do not correspond directly to complexes with a specific number of bound vapor molecules. Modeling in the subsequent section is used to compare measured inverse mobilities with theoretical predictions. Even without such modeling, it is evident that ion‐butanol complexes form readily, as ion inverse mobilities increase with increasing saturation ratio. Meanwhile *n*‐nonane does not adsorb onto any of the examined ions (at the examined saturation ratios). Qualitatively, this is in agreement with the droplet activation measurements of Winkler et al.,^39^ who found that smaller sized tungsten oxide seed ions could be used to initial droplet growth of *n*‐propanol vapors than could be used for n‐nonane. The increase in inverse mobility for butanol is most pronounced for the cations, and is inversely correlated with ion mass/size; though potassium is the smallest ion examined, upon introduction of butanol to the DMA it has the largest inverse mobility. Data hence reveal a clear sign dependency for butanol uptake, as well as a size dependency. The magnitude of increase in inverse mobility (more than a factor of 2 for the cations at saturation ratios greater than 0.10) is larger than what has been observed in prior studies where the vapor dopants were water^19^,^48^ and isopropanol.^31^,^32^ In Li and Hogan,^20^ ion‐vapor molecule complex formation was examined for (NaCl)_*n*_Na^+^ and (NaCl)_*n*_Cl^−^ ions with *n*‐butanol, ethanol, methyl ethyl ketone (1‐butanone), and toluene vapor molecules. Though such ions potentially dissolve/change structure during complex formation, similar findings were observed in this study. *n*‐Butanol, for a given solvent vapor concentration, led to the largest shifts in mobility for all sodium chloride cluster ions; inverse mobility shifts of more than a factor of 2 were observed. Adsorption of ethanol and methyl ethyl ketone led to increases in inverse mobility above 1.5 (in a similar vapor concentration range), while toluene, which, like *n*‐nonane, has a dipole moment below 0.5 D, led to minimal inverse mobility shifts.

Prior to more detailed model comparison, we remark that the initial inverse mobilities of the atomic ions in dry air are also within expectations. For the five ions examined, Table 1 lists the measured inverse mobility, as well as the predicted inverse mobility based upon the gas molecule scattering calculation approach described by Larriba and co‐workers.^49^, ^50^, ^51^, ^52^, ^53^ Calculations were performed modeling ions as spheres with radii equivalent to their ionic radii (noted in the table) and gas molecules as spheres with effective radii of 0.15 nm (based on prior measurements^54^,^55^). The ion‐induced dipole potential between ions and gas molecules was also considered (with a gas molecule polarizability 1.7×10^−30^ m^3^), and has a large impact on the predicted inverse mobilities of atomic ions. Calculations were performed modeling ion‐gas molecule collisions as completely elastic and specular (elastic hard sphere scattering, EHSS), as well as with the diffuse‐inelastic scattering model (diffuse hard sphere scattering, DHSS) of Larriba and Hogan.^50^ While prior studies^41^,^42^,^48^ reveal that gas molecule‐ion collisions in diatomic gases are neither wholly specular nor wholly diffuse (and are presumably a function of ion size, chemical composition, and the temperature), measurements should be bounded by EHSS and DHSS predictions. With the exception of the potassium cation, we find this to be true; measurements are bounded by the DHSS prediction as an upper limit the EHSS calculation as a lower limit. We suggest that the anomalously high inverse mobility of the potassium cation may be attributable to either the transient adsorption of contaminant vapor species during mobility analysis (this could shift mobilities by several percent for all examined ions); although efforts were made to minimize contamination of the system, completely removing all potential condensable species in ion mobility measurements has been shown to be difficult.^38^

**Table 1:** A summary of the measured and predicted (using diffuse hard sphere scattering and elastic hard sphere scattering models) inverse mobilities of atomic ions in air at atmospheric pressure and 304 K.^[a]^

Ion	Molecular mass [Da]	Ionic radius [Å]	Measured Inverse Mobility [Vs cm^−2^]	EHSS Prediction [Vs cm^−2^]	DHSS Prediction [Vs cm^−2^]
K^+^	39	1.52	0.471	0.310	0.434
Rb^+^	85	1.66	0.481	0.405	0.568
Cs^+^	133	1.81	0.486	0.453	0.591
Br^−^	80	1.82	0.445	0.349	0.450
I^−^	127	2.06	0.428	0.409	0.491

[a] Predictions were made considering the ion‐induced dipole potential between gas molecules and ions, and ions were modeled with the noted ionic radii.

### 2.2 Comparison to Classical Model Predictions of Vapor Uptake

Because we find non**‐**negligible mobility shifts in the presence of *n* **‐**butanol only, we compare a model of the mobility shift of ions in the presence of this vapor to measurements. Following the procedure developed in Oberreit et al.,^[18,19]^ the mobility of an ion (*K_S_*) exposed to vapor at saturation ratio *S* relative to its mobility in the absence of vapor (*K_0_*) can be computed using Equation (1):

(1) $$\frac{K_{S}}{K_{0}}=1+\Omega_{0}m_{0,b}^{1/2}\sum_{g=1}^{\infty }\left(\frac{P_{g}}{m_{g,b}^{1/2}\Omega_{g}}\right)$$

where *P_g_* is the probability an ion‐vapor molecule complex has *g* vapor molecules adsorbed to it at equilibrium (at the prescribed saturation ratio), *m*_*0,b*_ is the reduced mass of the bare ion and the bath gas, *mg*_*,b*_ is the reduced mass of the ion‐vapor molecule complex containing *g* vapor molecules, and $\Omega_{g}$ is is the collision cross section of ion‐vapor molecule complex considering collisions with the bath gas (with $\Omega_{0}$ the bare ion collision cross section). Equation (1) is developed accounting for the fact that if an ion equilibrates with the surrounding vapor during mobility measurement, the number of vapor molecules bound is not a constant, rather vapor molecules continually sorb and desorb from each complex, with the probability of finding an ion‐vapor molecule complex containing precisely *g* vapor molecules determined by the equilibrium binding coefficients for individual vapor molecules. Equation (1) neglects the influence of collisions between the dopant vapor and ion‐vapor molecule complex on drag/mobility. For the vapor pressures examined in this study we find this influence negligible, though note it has been shown in prior work to lead to a small, linear change (with vapor concentration) in the mobility of an ion in the absence of binding.^31^ Implementation of Equation (1) requires: a) Evaluation of *P_g_*, and b) Models for the collision cross‐sections of ion‐vapor molecule complexes.

For (a), a dimensionless equilibrium coefficient for the reaction [IV]_g‐1_ + [V] ⇌ [IV]_g_ (where [IV]_g_ is a ion‐vapor molecule complex with *g* vapor molecules bound, and [V] is the vapor molecule) can be defined balancing forward and reverse kinetic equations [Eq. (2)]:

(2) $$K_{eq,g}=\frac{k_{a,g-1}S}{k_{d,g}}exp\left(-\frac{\Delta E_{g}}{kT}\right)\;$$

In Equation (2), *k*_*a,g‐1*_ is the association rate coefficient for the noted reaction, *k*_*d,g*_ is the dissociation rate coefficient, *kT* is the thermal energy, and $\Delta E_{g}$ is the enthalpy difference between the ion‐vapor molecule complexes [IV]_g_ and [IV]_g‐1_ at saturation. *P_g_* can be expressed in terms of such equilibrium coefficients [Eq. (3a)a) and Eq. (3b)b)]:^19^

(3a) $$P_{g}=\frac{\prod_{j=1}^{g}K_{eq,j}}{1+\sum_{j=1}^{\infty }\prod_{i=1}^{j}K_{eq,i}}\;\;\;g\;\geq 1$$

(3b) $$P_{0}=\frac{1}{1+\sum_{j=1}^{\infty }\prod_{i=1}^{j}K_{eq,i}}$$

As in prior studies,^18^ the association and dissociation rate coefficients can be approximated as [Eq. (4a)a) and Eq. (4b)b)]:

(4a) $$k_{a,g-1}=\sqrt{\frac{8\pi kT}{m_{g-1,v}}}{\left(r_{g-1}+r_{v}\right)}^{2}\eta_{D}$$

(4b) $$k_{d,g}=\sqrt{\frac{8\pi kT}{m_{g,v}}}{\left(r_{g}+r_{v}\right)}^{2}$$

where *mg*_*,v*_ is the reduced mass for a vapor molecule and an ion‐vapor molecule complex (composed of *g* molecules), *r_g_* is the effective radius of an ion‐vapor molecule complex, *r_v_* is the effective radius of a vapor molecule, *kT* is the thermal energy, and $\eta_{D}$ is a dimensionless enhancement factor accounting for the influence of the ion‐dipole potential on ion‐vapor association (considered for butanol only). We approximate this factor using the equation [Eq. (4c)c)]:

(4c) $$\eta_{D}=1+C_{1}\frac{ze\mu_{D}}{4\pi \epsilon_{0}kT{\left(r_{g}+r_{v}\right)}^{2}}$$

where $\mu_{D}$ is the permanent dipole moment of the vapor molecule (1.66 D for *n*‐butanol), *ze* is product of the ion absolute charge state and the unit charge, and *C_1_* is a constant quantifying the fraction of time the dipole is aligned in the direction of the ion‐vapor molecule complex (taken to be 0.6 here). Nadytko and Yu^5^ have presented an alternative equation to Eq. (4c), which can be expressed as [Eq. (5)]:

(5) $$\eta_{D}=\sum_{n=0}^{\infty }\frac{{\left(\frac{ze\mu_{D}}{4\pi \epsilon_{0}kT{\left(r_{g}+r_{v}\right)}^{2}}\right)}^{2n}}{2n+1}$$

This expression is in reasonable agreement with Equation (4c) (i.e. within a factor of 2) for $\frac{ze\mu_{D}}{4\pi \epsilon_{0}kT{\left(r_{g}+r_{v}\right)}^{2}}$ <4, which is true for all ion‐vapor molecule complexes containing more than two vapor molecules in the present study. For larger values of $\frac{ze\mu_{D}}{4\pi \epsilon_{0}kT{\left(r_{g}+r_{v}\right)}^{2}}$ , the equation of Nadytko and Yu implies that the collision rate between vapor molecules and an ion increases with decreasing ion size,^56^ which is physically unreasonable, and predicts rates in excess of the *C_1_=1* in Equation (4c), which is the fully aligned dipole collision rate derived via the approach of Vasil'ev and Reiss.^57^ We therefore utilize Equation (4c) in all calculations presented here.

In Equations (4 a–c), the ion‐vapor molecule complex and vapor molecule are modeled as spheres. While prior work shows that a collision radius cannot be universally defined for a non‐spherical entity without considering the size and shape of its collision partner, (e.g. *r_v_* should depend upon *g*)^58^,^59^ we find that the spherical approximation does not strongly affect model predictions here. The ionic radii for each atomic ion, provided in Table 1, are used for r_0_ of ions with no vapor molecules bound, and ion‐vapor molecule complex radii calculated using the equation [Eq. (6)]:

(6) $$r_{g}={\left(r_{0}^{3}+gr_{v}^{3}\right)}^{1/3}$$

The radius of a butanol monomer was approximated from its molecular weight and bulk density, with a value of 3.3 Å.

**Figure 2:**
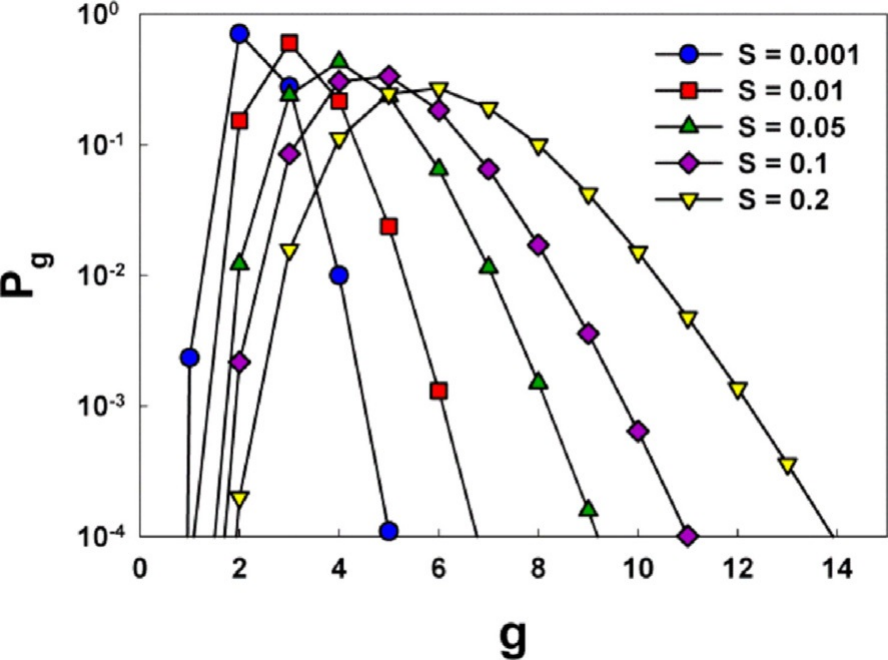
The probabilities (*P_g_*) for *g n*‐butanol molecules to be attached to a Rb^+^ ion at different saturation ratios, *S*, calculated using Kelvin–Thomson model.

Following the classical ion induced nucleation approach, $\Delta E_{g}$ can be written as the sum of two terms [Eq. (7a)a)]:

(7a) $$\Delta E_{g}=\Delta E_{g,K}+\Delta E_{g,T}$$

where the subscript *K* and *T* denote the Kelvin and Thomson contributions to the free energy, respectively. The Kelvin contribution can be written as [Eq. (7b)b)]:

(7b) $$\Delta E_{g,K}=\pi \sigma \left(r_{g}^{2}-r_{g-1}^{2}\right)$$

where σ is the surface tension/surface energy density of the ion‐vapor molecule complex (assumed to be the surface tension of the condensed vapor species, 0.024 J m^−2^). The Thomson contribution is [Eq. (7c)c)]:

(7c) $$\Delta E_{g,T}=\frac{{\left(ze\right)}^{2}}{8\pi \epsilon_{0}}\left(1-\frac{1}{\kappa }\right)\left(\frac{1}{r_{g}}-\frac{1}{r_{g-1}}\right)$$

where $\epsilon_{0}$ is the permittivity of free space, and κ is the complex dielectric constant (i.e. the condensed vapor dielectric constant, 17.8). These two terms combined serve as the basis classical ion‐induced nucleation theory predictions.^1^,^6^ $\Delta E_{g,K}$ is a positive term and quantifies the enthalpy barrier to growth, while $\Delta E_{g,T}$ is a negative term and quantities the barrier reduction brought about by the presence of charge. Sample Equation (3a, b) calculations of *P_g_* for *n*‐butanol with the Rb^+^ ion are provided in Figure 2. For each saturation ratio, a non‐negligible probability is found for multiple ion‐vapor molecule complexes, with the number of ion‐vapor molecule complex for which *P_g_* ≥0.01 increasing with increasing saturation ratio. This highlights the importance of accounting the sorption and desorption of vapor molecules from complexes during measurement; it is improbable that an ion would traverse the mobility analyzer with a constant number of vapor molecules bound to it.

For (*b*), following prior studies of the collision cross sections of nanometer scale ions,^49^,^50^ we approximate the ratio $\frac{\Omega_{g}}{\Omega_{0}}$ [Eq. (8)]:

(8) $$\frac{\Omega_{g}}{\Omega_{0}}={\left(\frac{r_{g}+r_{b}}{r_{0}+r_{b}}\right)}^{2}\frac{L\left(\Psi_{p,g}\right)}{L\left(\Psi_{p,0}\right)}$$

where *r_b_* is the effective radius of the bath gas (1.55 Å ) and $L\left(\Psi_{p,g}\right)$ describes the influence the induced‐dipole potential between bath gas molecules and the ion‐vapor molecule complex have on momentum transfer upon close approach. We calculate $L\left(\Psi_{p,g}\right)$ using the equation from Larriba and Hogan [Eqs. (9a), (9b) and (9a)c)]:^50^

(9a) $$L\left(\Psi_{p,g}\right)=1+0.329\Psi_{p,g}+0.089\Psi_{p,g}^{2}\Psi_{p,g}\leq 1$$

(9b) $$L\left(\Psi_{p,g}\right)=1+0.368\Psi_{p,g}-0.005\Psi_{p,g}^{2}\Psi_{p,g}>1$$

(9c) $$\Psi_{p,g}=\frac{\alpha_{p}z^{2}e^{2}}{8\pi \epsilon_{0}kT{\left(r_{g}+r_{b}\right)}^{4}}$$

Equation (9b) is only required for calculations involving the bare ion $\left(\Psi_{p,0}\right)$ , as it is only these ions for which $\Psi_{p,g}>1$ .

**Figure 3:**
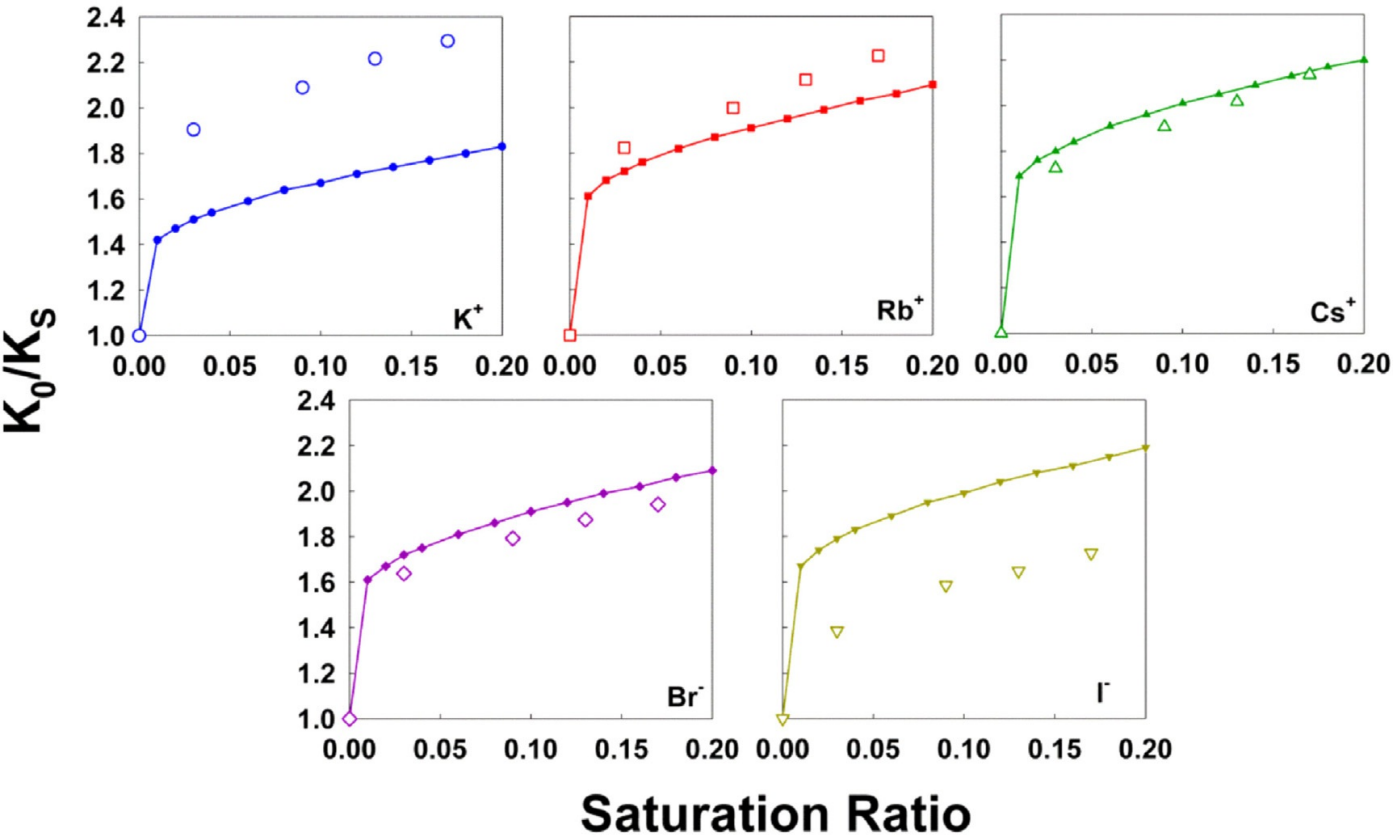
The ratio *K*_0_/*K_S_* as a function of *n*‐butanol saturation ratio for each of the examined ions. Open symbols: measured results. Closed symbols: Equation (1) predictions.

A comparison of Equation (1) predictions to measurements in terms of the ratio *K_0_/K_S_* (which increases with increasing saturation ratio) is shown in Figure 3. Experimental measurements and model predictions are in qualitative agreement; both show a rapid increase in *K_0_/K_S_* at low saturation ratios, followed by a more gradual increase. This is in contrast to Kelvin–Thomson predictions for larger ions;^18^,^19^ in these instances a small increase (below 10 %) in inverse mobility is predicted saturation ratios below 0.10, and then a drastic increase at higher saturation ratio (dependent upon the modeled activity coefficient). Additionally, for larger cluster ions, Kelvin–Thomson predictions have been found to be in poorer agreement with ion mobility‐mass spectrometry measurements than have simpler, Langmuir adsorption based models.^19^,^20^,^31^ However, for the Rb^+^_,_ Cs^+^, and Br^−^ ions examined here, model predictions are within 10 % of measured *K_0_/K_S_* values, suggesting that deviations observed in prior studies are at least partially attributable to the influence of cluster ion dissolution upon vapor sorption, as well as the influences of ion structure on sorption. Poorer agreement is observed for K^+^, and I^−^, the smallest and largest ions examined, with underprediction in the extent of mobility shift for K^+^ and overprediction for I^−^. Though the comparison is not shown, poorer agreement is also found between predictions and measurements of *n*‐nonane facilitated *K_0_/K_S_* shifts. The *n*‐nonane and *n*‐butanol model predictions primarily differ in that *n*‐nonane has a negligible dipole moment, and while this reduces the predicted extent of mobility ratio shift, it does not lead to model predictions of zero shift (as is observed for *n*‐nonane). Therefore, while Kelvin–Thomson predictions fit some results well, we caution against universal application of this model to describe vapor uptake, even by atomic ions.

**Figure 4:**
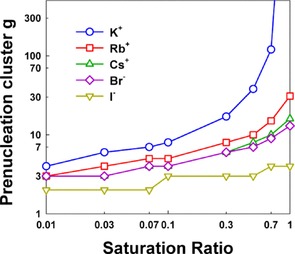
Predictions of the number of *n*‐butanol molecules in the largest stable prenucleation cluster based upon Equation (1), with the values for the surface density/surface energy density fit to measurements.

Without utilizing the bulk surface tension of *n*‐butanol in modeling and instead fitting the surface tension to measurements (minimizing the square error), for K^+^, Rb^+^, Cs^+^, Br^−^, and I^−^, we find effective surface tensions of 0.010, 0.022, 0.025, 0.027, and 0.044 J m^−2^, respectively, suggesting that the effective surface energy scales with ionic radius. The finding that smaller atomic ions uptake organic vapor to a greater extent (reflected in the smaller inferred surface tension) is in good agreement with the high pressure mass spectrometric measurements of Dzidic and Kebarle,^37^ who found that smaller alkali metal ions form larger complexes with water vapor than do larger ions, with Li^+^ exhibiting the largest (negative) enthalpy and Gibbs free energy change upon water vapor binding, and Cs^+^ the smallest enthalpy and Gibbs free energy change. However, the correlation between core ion size and extent of uptake does not appear to be universal for all vapor‐atomic ion combinations; high pressure mass spectrometry also reveals that the monovalent Sr^+^ ion would adsorb more water vapor than the Li^+^ ion,^36^ and Castleman et al.^35^ observed a weaker link between the extent of ammonia uptake and ion size. Using the effective surface energies noted above, the “prenucleation cluster” size can also be extrapolation as a function of saturation ratio, and is plotted in Figure 4. The prenucleation cluster size corresponds to the largest size at which Equation (2) predicted equilibrium coefficients are greater than unity. Following directly from the fit surface energies, the largest prenucleation clusters are predicted for K^+^ and the smallest for I^−^. Under subsaturated conditions, for Rb^+^, Cs^+^, and Br^−^, prenucleation ion‐vapor molecule complexes are anticipated to have between 3 and 30 vapor molecules bound; the largest of these clusters would be expected to have effective diameters near 1.4 nm, below the size detectable in condensation based particle detectors,^17^ but potentially with masses in excess of 1000 Da, which is larger than commonly encountered in ambient environments.^41^

## 3 Conclusions

We apply ion mobility‐mass spectrometry to examine the formation of ion‐vapor molecule complexes with seed ions of K^+^, Rb^+^, Cs^+^_,_ Br^−^, and I^−^ with *n*‐butanol and *n*‐nonane as the vapors, in air at atmospheric pressure near 304 K. Mobility shifts can be directly compared to model predictions based upon the Kelvin–Thomson equation, which is commonly invoked to predict ion induced nucleation rates. Based on these studies, we draw the following conclusions:

1. As was recently observed for sodium chloride cluster ions,^20^ the extent of mobility shift observed for atomic ions exposed to butanol is substantial; mobilities of all test ions are reduced by more than 40 % at butanol saturation ratios of 0.17. Conversely, *n*‐nonane does not appear to bind to atomic ions at similarly low saturation ratios, as even the transient binding of a single *n*‐nonane molecule would be lead to a detectable mobility shift. Ion mobility‐mass spectrometry experiments hence confirm a strong chemical dependency in the earliest stages of ion‐molecule complex formation in the vapor phase.
2. While the data do suggest that *n*‐butanol binds more strongly to cations than to anions (more uptake is observed for positively charged species), a clearer correlation is observed between the extent of mobility shift and ion size, with greater sorption observed for smaller atomic ions. The difference in the extent of sorption is large enough such that smaller atomic ions have lower mobilities at elevated butanol concentrations, that is, smaller ions actually form larger ion‐vapor complexes. This is consistent with high pressure mass spectrometry experiments with alkali metal seed ions and water vapor.^37^ However, it is not accounted for in Kelvin–Thomson equation predictions and suggests the binding energies of solvent molecules are not accurately predicted by this simple model alone.
3. Though the Kelvin–Thomson model does not accurately capture the influence of vapor molecule structure on complex formation (and prior work has shown it is difficult to modify this model to account for detailed chemical interactions),^4^ predicted mobility shifts based upon it are in reasonable agreement with observed shifts for Rb^+^, Cs^+^, and Br^−^ seed ions in the presence of *n*‐butanol. Therefore, despite what is noted in concluding remark 2, measurements here do suggest that for atomic seed ions, the Kelvin–Thomson model at least qualitatively captures features of ion‐vapor molecule complex formation. Complimentary experiments examining vapor sorption and nucleation upon these seed ions under supersaturated conditions^22^ will be useful to fully describe vapor uptake.

## Conflict of interest

*The authors declare no conflict of interest*.
